# Type I interferon production in myeloid cells is regulated by factors independent of *Ptpn22*

**DOI:** 10.1093/immhor/vlaf063

**Published:** 2025-10-09

**Authors:** Jenna R Barnes, Anam Fatima Shaikh, Alec M Bevis, Tammy R Cockerham, Robin C Orozco

**Affiliations:** Department of Molecular Biosciences, University of Kansas, Lawrence, KS, United States; Department of Molecular Biosciences, University of Kansas, Lawrence, KS, United States; Department of Molecular Biosciences, University of Kansas, Lawrence, KS, United States; Department of Molecular Biosciences, University of Kansas, Lawrence, KS, United States; Department of Molecular Biosciences, University of Kansas, Lawrence, KS, United States

**Keywords:** autoimmunity-associated allele, myeloid cells, *Ptpn22*, type I interferon

## Abstract

The immune regulatory gene *PTPN22* is expressed in all immune cells and encodes lymphoid protein (Lyp) in humans and the ortholog PEST domain–enriched phosphatase (PEP) in mice. The *PTPN22* alternative allele, 1858C>T, is expressed in 5% to 15% of the North American population and is strongly associated with the development of autoimmune disease while simultaneously capable of providing protection during viral infection and cancer. In murine models, significant progress has been made in elucidating the molecular mechanisms by which PEP and its pro-autoimmune variant (PEP-R619W) modulate T-cell function, yet their influence on non-T-cell pathways, such as antigen-presenting cell cytokine production, remains less defined. Previously, it was reported that PEP promotes type I interferon (IFN-I) production in dendritic cells (DCs) and macrophages following TLR4 stimulus. Here, we show that contrary to previous results, PEP and the PEP-R619W variant do not mediate IFN-I production in DCs and macrophages following exposure to LPS, 3p-hpRNA, or coronavirus mouse hepatitis virus strain A59. We attribute the prior findings to mouse strain–specific differences and conclude that factors independent of PEP may be regulating IFN-I production in these studies. We further show that PEP and its R619W variant distinctly modulate the production of TNF-α, IL-12, and IL-2 in DCs following LPS stimulus. Taken together, our results challenge the current understanding of the role of PEP during inflammation while providing new insight into how the PEP-R619W variant may alter myeloid cell function during disease.

## Introduction

Allelic variation in immune-regulatory genes can significantly impact disease outcomes.^1^^,^^2^ One variant of interest is the 1858C>T allele of the protein tyrosine phosphatase nonreceptor type 22 gene (*PTPN22*). *PTPN22* is constitutively expressed in all immune cells and encodes for the phosphatase lymphoid protein (Lyp) and its murine ortholog, PEST domain–enriched phosphatase (PEP). The *PTPN22* 1858C>T allele causes an amino acid substitution of arginine (R) to tryptophan (W) at position 620 in humans (Lyp-R620W). It is expressed in 5% to 15% of the North American population and is considered the highest non-HLA risk allele for the development of autoimmunity, including type 1 diabetes, rheumatoid arthritis, and systemic lupus erythematosus.^3–11^ To investigate the role of *PTPN22* and its autoimmunity-associated 1858C>T variant during inflammation, researchers often employ mouse models that lack expression of *Ptpn22* (PEP-null). However, the Lyp-R620W variant, or the murine equivalent PEP-R619W variant, does not cause a loss of protein expression, but rather altered function.^12^^,^^13^ Thus, using PEP-null models to investigate how the *PTPN22* 1858C>T autoimmunity-associated allele drives inflammation may not always be appropriate as PEP-null and PEP-R619W mice can have differing immune phenotypes at homeostasis and during disease.^14–20^

Previously, it was reported that PEP promotes type I interferon (IFN-I) production in dendritic cells (DCs) and macrophages following stimulus with bacterial lipopolysaccharide (LPS) or Poly(I:C), TLR4 and TLR3 agonists, respectively.^21^ These studies were performed using PEP-null mice available at The Jackson Laboratory (PEP-null JAX: strain number #028977) compared to C57BL/6J wild-type (WT) mice as the recommended controls per The Jackson Laboratory at that time. However, after these studies were published, the Jackson Laboratory discovered that the PEP-null JAX strain contain multiple single-nucleotide polymorphisms (SNPs) associated with the C57BL/6N mouse strain. These SNPs indicate the PEP-null JAX strain is on a mixed C57BL/6J; C57BL/6N background, despite reported backcrossing to the C57BL/6J strain.^22^ This is significant because the C57BL/6N SNPs present in this mouse model may impact immune responses such as IFN-I production. This poses concern that our current understanding of *Ptpn22* and how it regulates inflammation may have been confounded by mouse strain–specific genetic variation.

To enable a more rigorous investigation into defining how PEP and the PEP-R619W variant regulate inflammation and control for potential C57BL/6N-related genetic variation that could confound our results, our research team employs C57BL/6J CRISPR-Cas9–generated PEP-null and PEP-R619W mouse models, which have been maintained on a C57BL/6J background. Here, we employ these genetic models to determine if previous data regarding the impact of PEP on IFN-I production could be recapitulated and define if PEP and the PEP-R619W variant impacted the production of other cytokines in DCs. We report that neither PEP nor the PEP-R619W variant regulate IFN-β production in DCs or macrophages following LPS or RNA stimulation. However, we found that both PEP and PEP-R619W differentially modulate the production of other proinflammatory cytokines by DCs, including IL-2, IL-12, and TNF-α.

## Materials and methods

### Ethics

All animal studies were reviewed and approved by the University of Kansas Institutional Animal Care and Use Committee (IACUC) (protocol number: 278-01).

### Mice

Mice were bred and housed in specific pathogen–free general housing conditions at the University of Kansas (Lawrence, KS, USA). All animal studies were reviewed and approved by the University of Kansas IACUC (protocol number: 278-01). Both males and females ranging from 5 to 12 weeks of age were used in this study. C57BL/6J WT (PEP-WT) mice were originally purchased from The Jackson Laboratory, then bred and maintained in the University of Kansas Animal Care Unit. Ptpn22^−/−^ (PEP-null CRISPR) mice were generated using CRISPR-Cas9 technology on a C57BL/6J background, as described previously, by Dr Kerri Mowen and Dr Linda Sherman (Scripps Research Institute, La Jolla, CA, USA) and were gifted from Dr Sherman.^41^ Mice expressing the Ptpn22 proautoimmune allele (PEP-R619W) were generated using CRISPR-Cas9 technology on a C57BL/6J background using methods previously reported by Dr Kerri Mowen and Dr Linda Sherman (Scripps Research Institute) and were gifted from Dr Sherman.^18^^,^^19^^,^^41^ Approximately every 10 generations, PEP-null CRISPR and PEP-R619W mice are backcrossed to new C57BL/6J WT mice to reduce the likelihood that strain-specific SNPs have arisen and are driving a particular phenotype. Mice from the same parental pairs are not used in breeding pairs. B6.Cg-Ptpn22^tm2Achn^/J Ptpn22^−/−^ mice (PEP-null JAX) were gifted from Dr Won Jin Ho (John Hopkins University School of Medicine, Baltimore, MD, USA). These mice are available for purchase from The Jackson Laboratory (strain number #028977), and their generation has been previously described.^22^

### Bone marrow–derived macrophage culture

To generate bone marrow–derived macrophages (BMDMs), bone marrow cells were isolated from PEP-WT, PEP-null JAX, PEP-null CRISPR, and PEP-R619W mouse femurs. Following isolation, cells were cultured in RPMI medium supplemented with 10% FBS, 1% l-glutamine, and 1% penicillin-streptomycin with 50 ng/mL macrophage colony-stimulating factor (M-CSF) (Stemcell Technologies, Vancouver, BC, Canada) for 8 days. An additional 10 mL of M-CSF–containing media was added to BMDM cultures on day 3, and on day 8, differentiated cells were harvested, counted, and replated for further assays.

### Bone marrow–derived dendritic cell culture

Bone marrow cells were isolated from PEP-WT, PEP-null, and PEP-R619W mouse femurs. Following isolation, bone marrow cells were cultured in advanced DMEM containing 10% FBS, 1% penicillin-streptomycin, 1% l-glutamine, and 100 ng/mL FMS-like tyrosine kinase 3 ligand (FLT3-L; Stemcell Technologies) for 8 days. On day 8, FLT3-L–differentiated DCs were harvested, counted, and replated for further assays.

### Myeloid cell stimulation

#### BMDM stimulation

BMDMs were plated in a 96-well plate at a density of 5 × 10^4^ cells per well. For infection, cells were inoculated with mouse hepatitis virus strain A59 (MHV A59) at a multiplicity of infection (MOI) of 0.1. The plate was incubated for 1 hour at 37 °C with shaking every 10 minutes to ensure even distribution of the virus. Following the 1-hour incubation, the plate was centrifuged at 300 × *g* for 10 minutes to pellet the cells. The supernatant containing the unadsorbed virus was carefully discarded. The cells were then resuspended in fresh media and incubated at 37 °C for 18 hours to allow for viral replication. After the 18-hour incubation, the cell culture supernatant was collected and stored at −80 °C for subsequent IFN-β ELISA.

#### Bone marrow–derived dendritic cell stimulation

Bone marrow–derived dendritic cells (BMDCs) were resuspended and plated at 1 × 10^6^ cells/mL in advanced DMEM (Gibco) containing 10% FBS, 1% penicillin-streptomycin, and 1% l-glutamine. The cells were stimulated with either LPS or 5′ triphosphate hairpin ribonucleic acid (3p-hpRNA). Cells stimulated with LPS (500 ng/mL) (Sigma) were incubated overnight (16 to 18 hours). The supernatant was collected and stored frozen at −80 °C for future assays. Cells stimulated with 3p-hpRNA (2 ng/mL) complexed with LyoVec (InvivoGen) were incubated overnight (16 to 18 hours). The supernatant was collected and stored frozen at −80 °C for future assays.

### ELISA

Levels of IFN-β, TNF-α, IL-12 (p70), and IL-2 in cell culture supernatant were measured using ELISA Kits (IFN-β, PBL Assay Science; TNF-α, IL-12, and IL-2, BioLegend) according to the manufacturer’s instructions. Absorbance measured by a BioTek EON microplate reader at 450 and/or 570 nm.

### qRT-PCR

RNA was extracted from 6- to 8-week-old PEP-WT and PEP-null mouse splenocytes using TRIzol according to the manufacturer’s instructions (Invitrogen). cDNA was synthesized using the High-Capacity cDNA Reverse Transcription Kit (Applied Biosystems), according to the manufacturer’s instructions. Real-time PCR was performed on a Quant Studio 3 system using PowerTrack SYBR Green Master Mix (Applied Biosystems). Each reaction was measured in duplicate, and data were normalized to the expression levels of the housekeeping gene *Gapdh*. Primers for the genes were as follows: *Gapdh*: forward primer 5′-ACGACCCCTTCATTGACCTC-3′ and reverse primer 5′-ACTGTGCCGTTGAATTTGCC-3′; *Ptpn22*: forward primer 5′-AGCTGATGAAAATGTCCTATTGTGA-3′ and reverse primer 5′-GTCCCACTGCATTCTGGTGA-3′.

### Statistical analysis and graphing

All statistical analysis was performed using GraphPad Prism software. The type of statistical test is listed in the figure legends. Data were considered statistically significant if *p* < 0.05. Graphs were made in GraphPad Prism software. Figure legends indicate if data shown are pooled from multiple studies or are from a representative study.

## Results

### Strain-specific differences impact IFN-β production in PEP-null BMDCs

**Figure 1.**
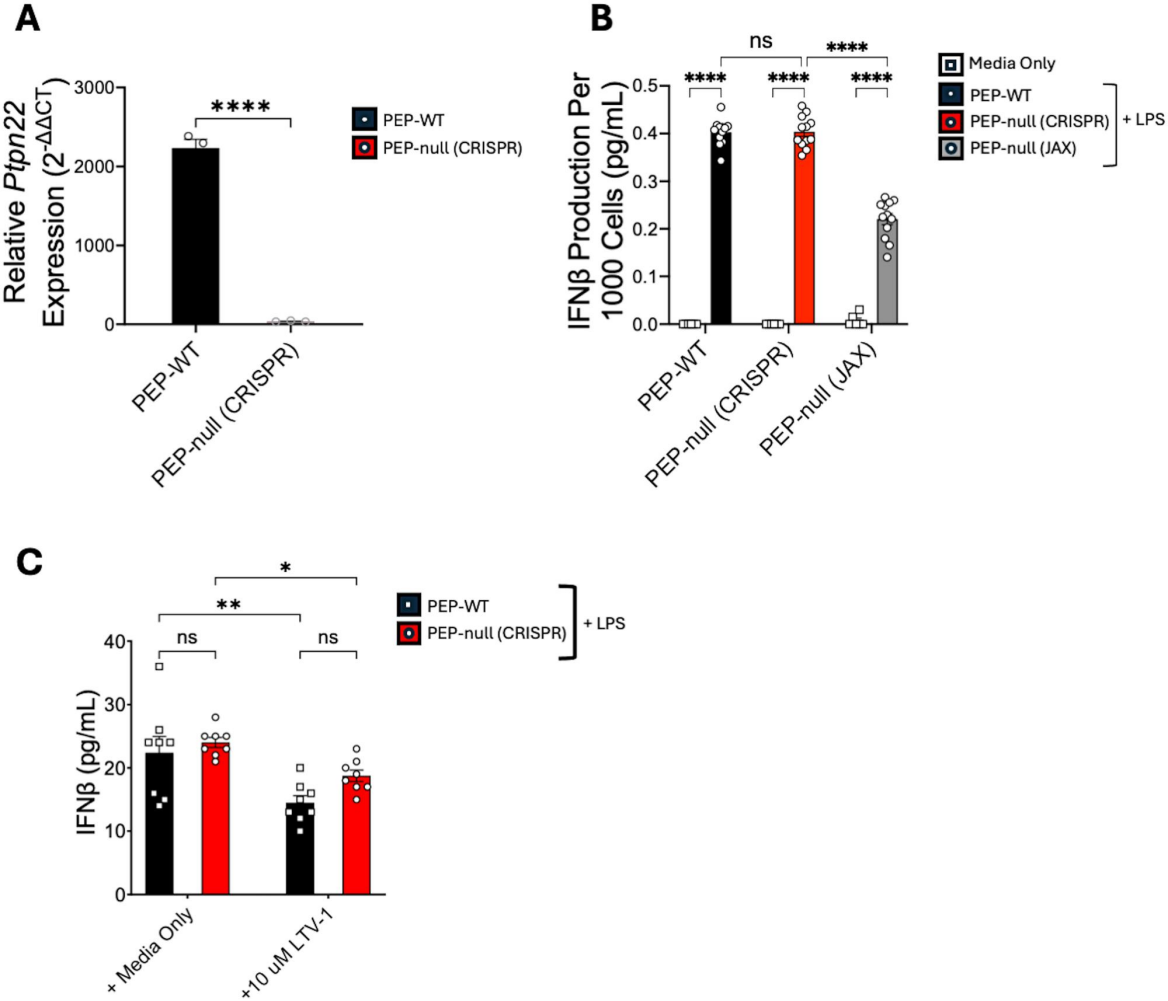
Strain-specific differences impact IFN-β production in PEP-null BMDCs. (A) Gene expression values of *Ptpn22* relative to *Gapdh* were determined by qRT-PCR for naïve PEP-WT (black) and PEP-null (CRISPR) (red) splenocytes; each dot represents one mouse. (B) FLT3-L BMDCs were cultivated and differentiated from C57BL/6J PEP-WT (black), CRISPR-Cas9–generated PEP-null (red), and PEP-null mice from The Jackson Laboratory (JAX stock #028977) (gray). Cultures were incubated with LPS (500 ng/mL) for 18 hours. The supernatant was used to determine IFN-β concentration (ELISA). (C) PEP-WT and PEP-null (CRISPR) BMDMs were incubated with LPS (500 ng/mL) and 10 µM LTV-1 for 18 hours. The supernatant was used to determine IFN-β concentration (ELISA). *P < 0.01, **P < 0.001, ****P < 0.0001; ns, not significant. Unpaired *t*-test (A) or 2-way ANOVA with Tukey post hoc analysis (B, C). Data are pooled from 2 independent experiments. Each dot represents a biological replicate.

Previously, it was shown that PEP-null BMDCs and BMDMs produce reduced amounts of IFN-I when stimulated with bacterial LPS or Poly(I:C), TLR4 and TLR3 agonists, respectively.^21^ However, the PEP-null JAX strain was discovered to have multiple SNPs associated with a mixed genetic background, posing the concern that our current understanding of the role of PEP in regulating IFN-I production may have been influenced by mouse strain–specific differences, such as genetic variation. Using our C57BL/6J CRISPR-Cas9–generated PEP-null model (PEP-null CRISPR), we first set out to determine if previous data regarding the impact of PEP on IFN-I production could be recapitulated. Using qRT-PCR, we confirmed that our PEP-null CRISPR mice do not express quantifiable amounts of *Ptpn22* mRNA (Fig. 1A). Then, using FLT3-ligand differentiated BMDCs from C57BL/6J PEP-WT, PEP-null CRISPR, and PEP-null JAX mice, we measured for IFN-β production following LPS stimulation. While IFN-β concentration was comparable between PEP-WT and our PEP-null CRISPR mice, BMDCs from the commercial PEP-null JAX strain showed significantly less IFN-β production (Fig. 1B). These data successfully recapitulate prior results, but through the use of our CRISPR-Cas9–generated PEP-null model, we observe that PEP does not promote IFN-β production. The differences between the PEP-null JAX and PEP-null CRISPR strains suggest that differences associated with the C57BL/6N background, rather than *Ptpn22* deficiency alone, may account for the previously reported phenotype.

In addition to the PEP-null JAX model, studies have also utilized chemical inhibition of PEP to evaluate its role in IFN-I production and activity.^21^^,^^23^ However, varying results regarding PEP inhibitors and their specificity have been reported.^24^ To evaluate the specificity of the PEP inhibitor LTV-1 during IFN-I production, we stimulated PEP-WT and PEP-null CRISPR BMDMs with LPS in the presence or absence of LTV-1 to inhibit the enzymatic activity of PEP. We observed reduced IFN-β production in both PEP-WT and PEP-null CRISPR BMDMs treated with LTV-1, but no significant differences in IFN-β production between the 2 genotypes (Fig. 1C). These data suggest that the reduction in IFN-β production is due to off-target effects of LTV-1, rather than inhibiting PEP. Given the potential confounding factors associated with the PEP-null JAX mice and off-target effects of LTV-1, we proceeded with our studies to define the impact of PEP and the PEP-R619W variant on inflammation with only our C57BL/6J CRISPR-generated mouse strains.

### PEP and PEP-R619W do not mediate IFN-β production in myeloid cells

**Figure 2.**
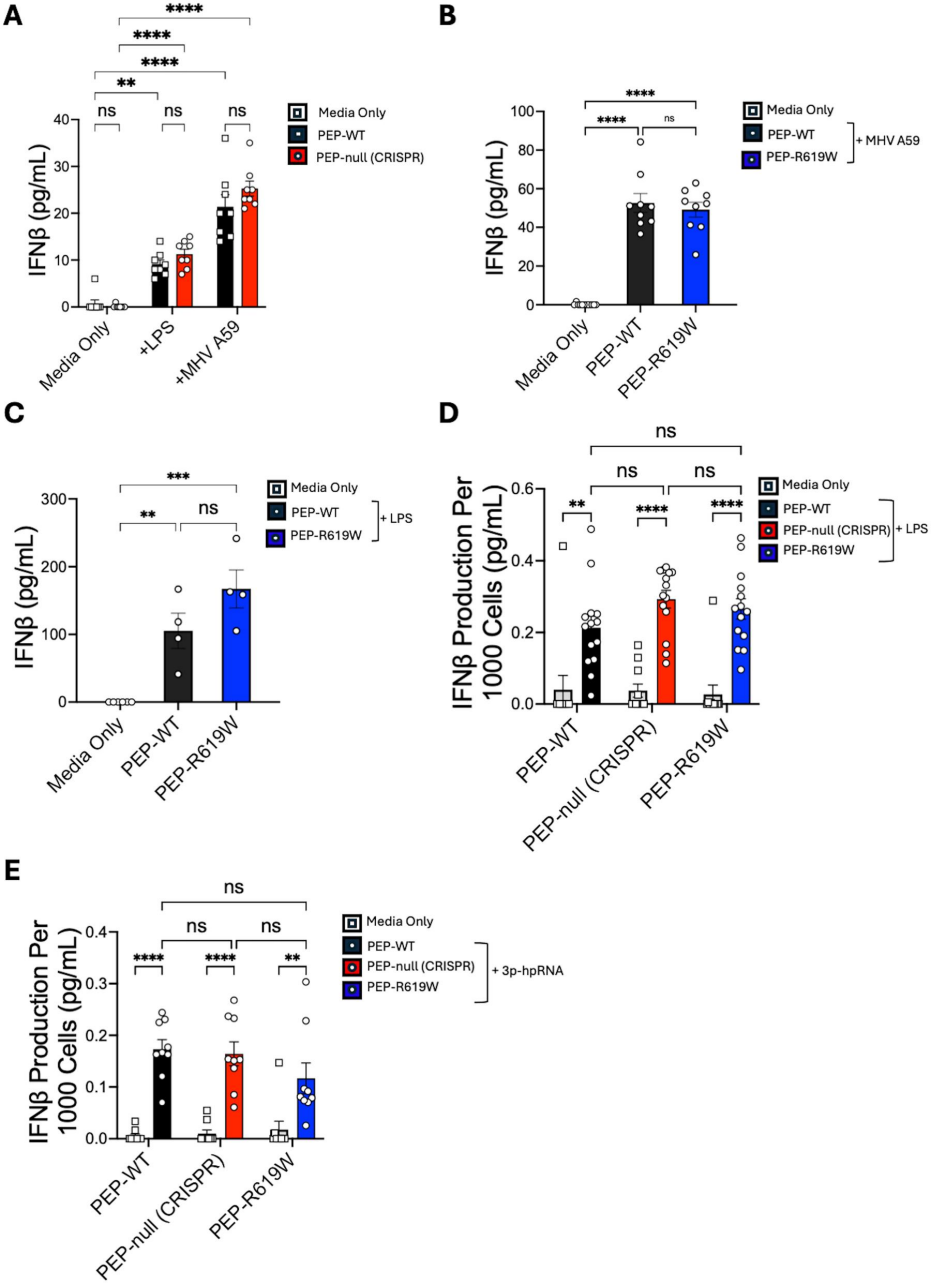
PEP and PEP-R619W do not mediate IFN-β production in myeloid cells following LPS, MHV A59, or viral RNA stimulation. (A) C57BL/6J PEP-WT (black, squares), or CRISPR-Cas9–generated PEP-null (red, circles) BMDMs were cultivated and then stimulated with LPS (500 ng/mL) or infected with MHV A59 (MOI = 0.1) for 18 hours. (B, C) C57BL/6J PEP-WT (black, circles) and CRISPR-Cas9–generated PEP-R619W (blue, circles) BMDMs were cultivated and then stimulated with MHV A59 (MOI = 0.1) (B) or with LPS (500 ng/mL) (C) for 18 hours. (D, E) FLT3-L BMDCs were cultivated from C57BL/6J PEP-WT, PEP-null, and PEP-R619W mice. Cultures were incubated with LPS (500 ng/mL) (D) or 3p-hpRNA (2 ng/mL) (E) for 18 hours. The supernatant was used to determine IFN-β concentration (ELISA). **P < 0.001, ****P < 0.0001; ns, not significant. Two-way ANOVA with Tukey post hoc analysis (A, D, E) or one-way ANOVA (B, C). Data are representative of 2 independent experiments (A) or pooled from 3 independent experiments (B–E). Each dot represents a biological replicate.

Our initial results showed comparable IFN-β production between PEP-WT and PEP-null CRISPR BMDCs following TLR4 stimulation via LPS. However, beyond TLRs, additional pattern recognition receptors such as RIG-I like receptors (RLRs) induce large quantities of IFN-I in response to viral infection.^25–27^ Further, it is unknown if the autoimmunity-associated PEP-R619W variant, which alters protein function rather than expression, uniquely affects IFN-I production compared to PEP-WT. To determine if the PEP-R619W variant either phenocopies the complete loss of PEP or distinctly alters IFN-I production following RLR activation, we cultivated PEP-WT, PEP-null CRISPR, and PEP-R619W BMDMs and then stimulated them with LPS or infected with the murine coronavirus MHV A59, a melanoma differentiation–associated protein 5 (MDA5) agonist.^27^ There were no differences detected in IFN-β production between PEP-null CRISPR and PEP-WT BMDMs following LPS exposure or MHV A59 infection (Fig. 2A). There were also no difference detected in IFN-β production between PEP-WT and PEP-R619W BMDMs following MHV A59 infection (Fig. 2B) or LPS exposure (Fig. 2C). These results suggest that PEP does not promote IFN-I production in BMDMs following TLR4 or MDA5 activation.

Next, we generated FLT3-L BMDCs from C57BL/6J PEP-WT, PEP-null CRISPR, and PEP-R619W mice and stimulated them with LPS or 3p-hpRNA, a RIG-I agonist. We detected no significant differences in IFN-β production between all 3 genotypes (PEP-WT, PEP-null CRISPR, and PEP-R619W) following either LPS or 3p-hpRNA stimulation (Fig. 2D, E). Taken together, these results indicate that neither PEP nor the PEP-R619W variant modulate IFN-β production in DCs mediated by TLR4 or RIG-I activation.

### PEP and the PEP-R619W variant distinctly modulate the production of proinflammatory cytokines in DCs

Our findings show that neither PEP nor the PEP-R619W variant impact IFN-I production in DCs and macrophages during TLR4, MDA5, or RIG-I stimulation. However, DCs produce a variety of other cytokines that can promote widespread inflammation or influence T-cell activation and differentiation. Prior studies show that PEP-null and the PEP-R619W variant enhance T-cell function through both T-cell–intrinsic and T-cell–extrinsic mechanisms during viral infection.^19^^,^^28^^,^^29^ Previous studies that have investigated the role of PEP and its variant on cytokine production in myeloid cells have reported varied findings.^21^^,^^30^^,^^31^ Using PEP-R619W knock-in mouse models, one group reported increased IL-12 production in granulocyte-macrophage colony-stimulating factor (GM-CSF)–differentiated PEP-R619W BMDCs post–LPS exposure.^30^ Another group reported that GM-CSF–differentiated PEP-null JAX BMDCs exhibited enhanced secretion of IL-6, IL-8, and TNF-α compared to PEP-WT BMDCs following treatment with muramyl dipeptide.^31^ They also reported that the loss of PEP reduced while the PEP-R619W variant increased IL-1β secretion by BMDCs following ultra-pure LPS exposure.^20^ However, a different group later reported no differences in TNF-α, IL-1β, or IL-12 production following treatment with LPS between FLT3-L–differentiated PEP-WT and PEP-null JAX BMDCs.^21^ These contrary results align with previous reports of PEP-null and PEP-R619W mice exhibiting different phenotypes but could also be attributed to the usage of different mouse models, type of stimulus, and selected assays. Thus, using our CRISPR-Cas9–generated mouse models, we wanted to know if PEP and the PEP-R619W variant regulated the production of other inflammatory cytokines relevant to modulating T-cell function.

**Figure 3.**
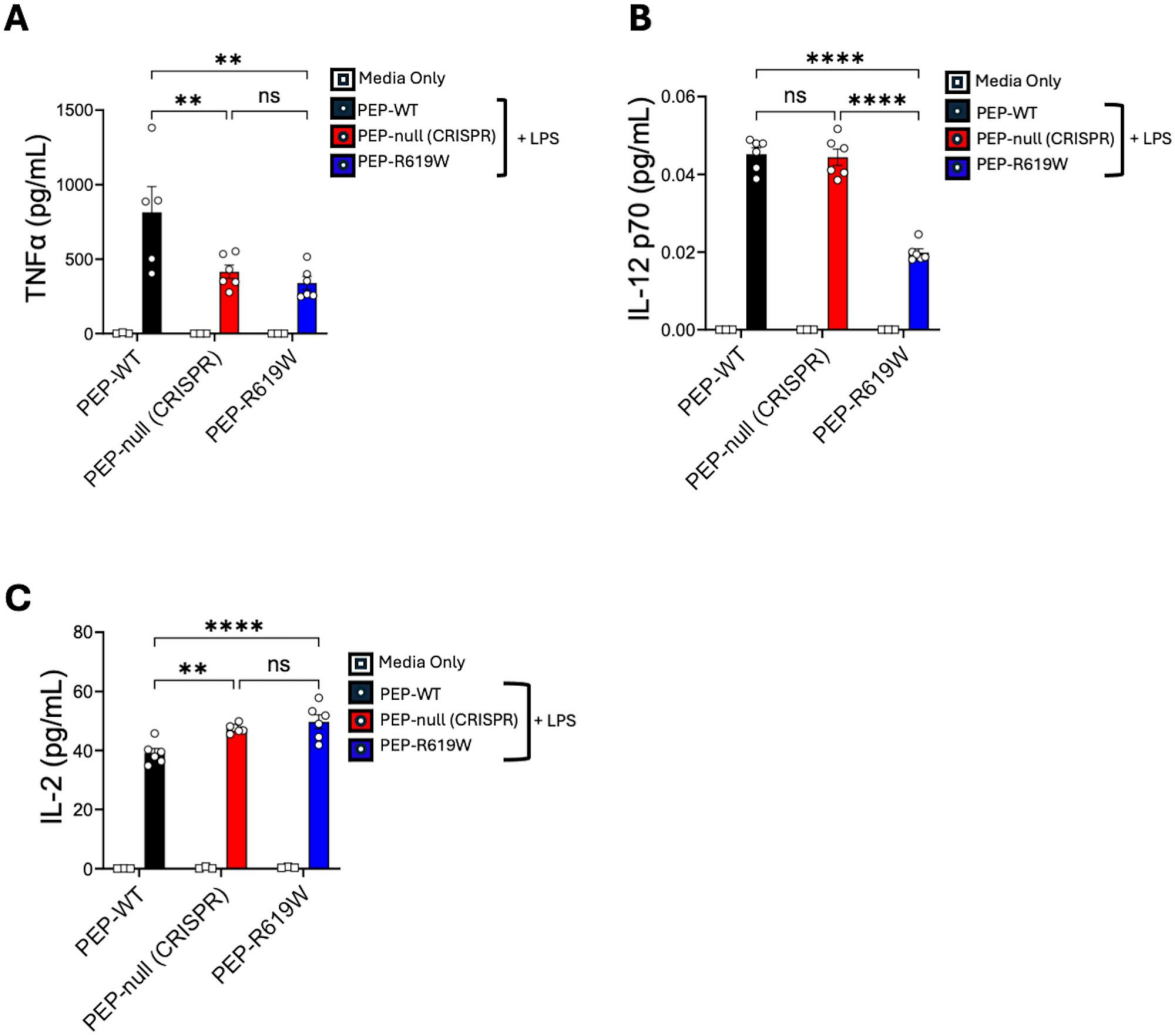
PEP and the PEP-R619W variant distinctly modulate the production of proinflammatory cytokines in DCs. FLT3-L BMDCs were cultivated from C57BL/6J PEP-WT (black), CRISPR-Cas9–generated PEP-null (red), and PEP-R619W (blue) mice. Cultures were incubated with LPS (500 ng/mL) for 18 hours. The supernatant was used to determine TNF-α (A), IL-12 (B), and IL-2 (C) concentrations (ELISA). **P < 0.01, ****P < 0.0001; ns, not significant. Two-way ANOVA with Tukey post hoc analysis. Data are representative of 2 independent experiments. Each dot represents a biological replicate.

To accomplish this, we again used FLT3-L–differentiated BMDCs from PEP-WT, PEP-null CRISPR, and PEP-R619W mice. BMDCs were stimulated with LPS, and the supernatant was collected and analyzed for TNF-α, IL-12 (p70), and IL-2 production. We found that compared to PEP-WT BMDCs, TNF-α production was reduced in both PEP-null CRISPR and PEP-R619W BMDCs (Fig. 3A). Additionally, we observed a reduction in IL-12 production by PEP-R619W BMDCs compared to PEP-WT. However, there was no difference detected in IL-12 production between PEP-null CRISPR and PEP-WT BMDCs (Fig. 3B). Finally, we observed increased IL-2 production by both PEP-null CRISPR and PEP-R619W BMDCs compared to PEP-WT BMDCs (Fig. 3C). Taken together, these findings suggest that PEP and the PEP-R619W variant distinctly modulate cytokine production in DCs.

## Discussion

The *PTPN22* 1858C>T allele is strongly associated with the development of autoimmunity, but the murine equivalent mutation has also been shown to be protective during viral infection and cancer.^18^^,^^19^^,^^32^ While the impact of the PEP-R619W variant is well-defined in T cells, the molecular mechanisms by which PEP and its variant impact the function of other immune cells, such as the ability of APCs to produce proinflammatory cytokines, remain less understood. Previously, it was suggested that PEP promotes IFN-I production in macrophages and dendritic cells.^21^ However, these data were generated using PEP-null mice on a C57BL/6J; C57BL/6N mixed genetic background, posing the concern that our current understanding of the role of PEP during IFN-I production may have been influenced by the C57BL/6N SNPs present in the PEP-null JAX mouse model. Using our CRISPR-Cas9–generated PEP-null model, we first set out to determine if previous data regarding the impact of PEP on IFN-I production could be recapitulated. While the PEP-null JAX BMDCs had reduced IFN-I production, we did not detect a difference between our PEP-null CRISPR BMDCs and C57BL/6J PEP-WT BMDCs (Fig. 1B). These strain-specific differences in IFN-I production recapitulate previous findings, while also revealing that strain-specific differences, likely genetic variation between C57BL/6J and C57BL/6N mice, in the PEP-null JAX mice may have influenced the previously observed phenotype. This interpretation is further supported by reports of reduced inflammatory responses during viral infection in C57BL/6N mice compared to C57BL/6J mice.^33^^,^^34^ However, our study results do not address why PEP-null JAX BMDMs engineered to overexpress the human protein Lyp have restored IFN-β production.^21^ Identifying the mechanism that drives the reduced IFN-β production observed in the C57BL/6J; C57BL/6N PEP-null JAX mice but not in the PEP-null CRISPR mice will require further investigation. Regardless, we are not the first to comment on or identify discrepancies regarding previous data reported on the role of PEP during IFN-I production in myeloid cells. However, these prior publications have largely focused on differences identified in human samples, as well as in the context of systemic lupus erythematosus.^35–39^ Thus, to the best of our knowledge, we are the first to show in a mouse model that there may be strain-specific differences confounding the interpretation of how/if PEP regulates cytokine production in DCs and macrophages.

Next, when evaluating the specificity of the PEP inhibitor LTV-1 during IFN-I production in BMDMs, we found reduced IFN-β production in both PEP-WT and PEP-null CRISPR macrophages treated with LTV-1 (Fig. 1C). These findings suggest that the observed reduction of IFN-β may not be due to the deficiency of PEP nor the inhibition of its enzymatic function, but rather because of nonspecific effects of LTV-1. Taken together, our findings reveal additional confounding variables that could impact the results of studies that aim to discern the role of PEP during inflammation through the use of either genetic models or chemical inhibition. Our observations add to the numerous conflicting findings already identified in the field regarding PEP, PEP-R619W, and their effects on cytokine production.^20^^,^^21^^,^^30^^,^^31^ These differences likely stem from genetic variation present in both human and animal studies, as well as the utilization of diverse experimental approaches including overexpression, transgenic models, knockdown techniques, and chemical inhibition. Each of these systems offers distinct advantages yet also varying limitations, yielding inconsistent results. To address this lack of clarity, our laboratory employs CRISPR-Cas9–generated PEP-null and PEP-R619W mouse models on a C57BL/6J background. By using this model, we can precisely evaluate the mechanisms by which PEP and the PEP-R619W variant drive inflammation while minimizing confounding genetic variables.

Using our PEP-null CRISPR and PEP-R619W mice, our results indicate that IFN-β production by DCs and macrophages is not promoted by PEP or the PEP-R619W variant following TLR4, MDA5, or RIG-I stimuli (Fig. 1B, Fig. 2A–C). Although our findings indicate that PEP and PEP-R619W do not promote IFN-β production in myeloid cells, we found that PEP and the PEP-R619W variant distinctly modulate the production of other proinflammatory cytokines. More specifically, we found that compared to PEP-WT, TNF-α production was reduced in both PEP-null CRISPR and PEP-R619W DCs (Fig. 3A). Additionally, we observed a reduction in IL-12 production by PEP-R619W DCs compared to PEP-WT, but no significant change in the production of IL-12 was detected in the PEP-null CRISPR BMDCs when compared to PEP-WT BMDCs (Fig. 3B). Previously it was reported that PEP-R619W GM-CSF–differentiated BMDCs have increased IL-12 production compared to PEP-WT.^30^ These conflicting findings may be due to a variety of factors such as differences in the PEP-R619W mouse models, LPS dosage, and/or functional differences between FLT3-L–differentiated and GM-CSF–differentiated BMDCs. Finally, we observed increased IL-2 production by both PEP-null CRISPR and PEP-R619W BMDCs compared to PEP-WT. Since DC-derived IL-2 can stimulate the activation of T cells, it is possible that the increased IL-2 produced by PEP-null CRISPR and PEP-R619W DCs may be a contributing factor to the enhanced T-cell function observed during viral infection.^19^^,^^28^^,^^40^

Altogether, our findings challenge the current understanding of PEP while providing new insight into how the PEP-R619W variant is capable of simultaneously promoting autoimmunity and conferring protection against persistent viral infection and cancer. While our results reveal that changes in IFN-I production are not a key factor in how PEP regulates inflammation as previously regarded, our data indicate that PEP and the PEP-R619W variant have a novel role in modulating the production of other cytokines by DCs, which are critical for a variety of inflammatory processes. Future studies will need to evaluate how changes mediated by PEP and the PEP-R619W variant in DCs and other antigen-presenting cells are relevant in order to accurately define the broad consequences they have on disease pathogenesis.

## Data Availability

All data necessary to interpret results are presented in this manuscript. Raw data will be made available upon reasonable request to the corresponding author.
